# The VODAN IN: support of a FAIR-based infrastructure for COVID-19

**DOI:** 10.1038/s41431-020-0635-7

**Published:** 2020-05-06

**Authors:** Barend Mons

**Affiliations:** Human Genetics, LUMC, Leiden, The Netherlands

**Keywords:** Diseases, Infectious diseases

## The VODAN core consortium

### Origin and first weeks

The Virus Outbreak Data Network (VODAN) Implementation Network (IN)^1^ was conceived to kick-start a ‘community of communities’ that could design and rapidly build a truly international and interoperable, distributed data network infrastructure that supports evidence-based responses to the viral outbreak. The IN has a longer-term goal to reuse the resulting data and service infrastructure, also for future outbreaks. As a GO FAIR IN, VODAN will restrict itself to projects that are directly associated with FAIR data [1] and services relevant to COVID-19. This also means that VODAN will not concern itself with projects purely aimed at studying, for instance, transmission and severity genetics, experimental drug development, vaccine development or actual clinical interventions currently executed to control the epidemic. Only insofar as such activities are in need of access to, and processing of, FAIR data, VODAN partners can add FAIR data and services value.

Early on in the process, CODATA^2^, the committee of the International Science Council^3^, the Research Data Alliance (RDA)^4^ and the World Data system^5^ now also operating in joint actions under the name ‘Data Together’^6^, reviewed the initial actions of VODAN and issued a joint statement^7^ of support. At the request of the European Commission, RDA established a Working Group^8^, which is also co-supported by the Data Together organisations.

IN–IN collaboration: As a typical, open, GO FAIR Implementation Network, VODAN is open to all parties, public or private, who commit to follow the FAIR guiding principles and who explicitly wish to prevent vendor lock-in (see GO FAIR rules of engagement: https://www.go-fair.org/resources/rules-of-engagement/). The VODAN partners rely on the communities already established in other GO FAIR INs, including the Personal Health Train^9^, IN-Africa^10^, the Data Stewardship Competence Centers^11^, C2CAMP^12^ (for core infrastructure), Chemistry^13^, CO-OPERAS^14^ (for SSH data), DISCOVERY^15^ (search, visualisation) and FAIR StRePo^16^ (standards and policies).

**Fig. 1:**
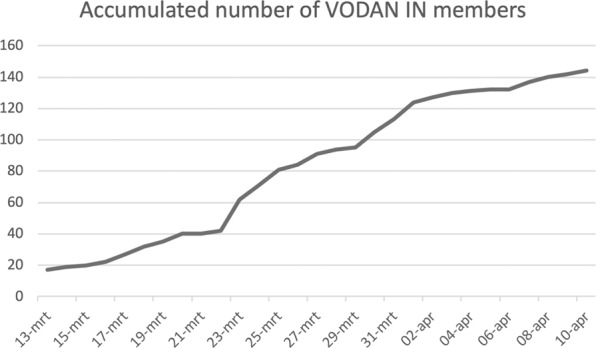
Accumulated number of VODAN IN members. The rapid growth of partners in the first weeks of the virus Outbreak Data Network.

Since its approval as an IN on March 13, the number of partners in VODAN increased much faster than in regular INs and on April 10, the network already approached 150 partners (Fig. 1) This indicates a perceived need for community based networking.

The current VODAN partners include both public and private parties who united around the notion that FAIR data will be needed in many aspects of an adequate response to the current, as well as to future infectious disease outbreaks.

In VODAN, we clearly distinguish between ‘established knowledge’ (EK) based on the published literature, databases and public datasets, and what we call ‘Real World Observations’ (RWO), made on actual situations in real time. The main challenge of VODAN is on the latter class of data, as many other public as well as private organisations are already dealing with FAIR-enabled public databases and applications (such as for instance ELIXIR, BBMRI, EBI, NLM, Euretos). Those organisations that work on EK bases and biomedical research results are obviously candidate ‘data stations’ in the Personal Health Train sense and these are welcome to the VODAN IN and to format their assets also according to the FAIR principles.

Rationale: Why is access to the necessary RWO, high quality, data on the current epidemic so difficult?

First, there is the established ‘GDPR’ issue of citizen/patient privacy and this means that the RWO cannot just be ‘open’. VODAN will also demonstrate the difference between ‘FAIR’ and ‘open’ by combining restricted data with opne data [2]. Second, this epidemic is particularly highly politicised, and there is little chance that countries (or even institutions) will ‘share their RWO data’ in the classical way, i.e. by ‘sending’ them to a central data warehouse, not even at WHO. A third issue to address is that, like in the earlier HIV epidemic, there will be so much money forthcoming for research and experimental interventions that there will be an enormous variety of overlapping, and even redundant projects and consequently a wave of data, of which a significant portion will be of questionable quality. This means that we need a strong emphasis on distributed analytics as well as on vetting and annotation of data, for which we need (expert) distributed annotation in near-real time. This can only be done with machine-assistance and through (expert) community-based massive annotation.

These considerations are not unique to the current COVID-19 epidemic and the mitigation of these problems is at the core of high-quality, reproducible and peer reviewed science leading to evidence-based interventions. In other words, a modern ‘epidemic-ready data and services infrastructure’ would have to address all these issues very effectively. It was from this perspective that we came up with the boundary conditions for a ‘COVID-19 data and services infrastructure’ in the common Data Together statement.

## A global, equitable and rapid-response data infrastructure for the COVID-19 and future epidemics

Much COVID-19-related RWO data have political or institutional sensitivities, some have personal components. Sensitive and personal data cannot be ‘open’ and the sensitive and personal data associated with COVID-19 cannot leave the country, and in most cases, the institution that controls them. Such data can only be accessed partially and in controlled circumstances, and for this to be achieved, the data must be FAIR. We need, therefore, to make a rigorous choice in favour of FAIR (as opposed to always Open) while continuing to emphasise the policy: As open as possible, as closed as necessary.

In such circumstances (and many others) a centralised, data warehousing approach is not fit for purpose, if possible at all. As the data are de facto distributed, rich FAIR metadata is necessary to enable controlled, computational access for analysis or visualisation.

Very large quantities of data are being generated in relation to the pandemic. There are also significant challenges in ensuring data quality, as well as risks of false and misleading information being disseminated as ‘fact’. This poses challenges for science and society: there should be a mechanism to mitigate such dangers.

Consequently, we need to facilitate and further enhance infrastructure and methods for ‘distributed deep learning’, and make sure that the algorithms and services on which such approaches are based can work effectively with FAIR metadata and, where possible, with FAIR data, with emphasis on the policy: as distributed as possible, as centralised as necessary.

The current circumstances necessitate urgent development, in interfaces with many services and components, of a ‘community annotation system’ that enables objective assessment of new claims and information. Experts, funders and advisory groups can then rely on a vast community of trusted experts to review new and existing claims relevant for COVID-19 interventions.

It is essential to avoid that any particular party can monopolise the FAIR ecosystem and its applications. Therefore, a quality control and a minimal certification scheme for all components must be in place as part of the effort.

**Fig. 2:**
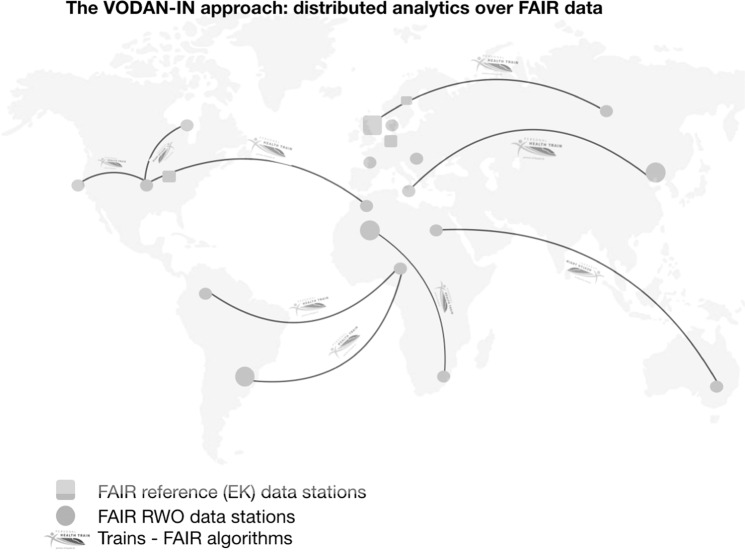
Artist impression of the envisioned distributed learning environment based on PHT technology approaches.

Recognising this urgent need and the opportunity for an accelerated development of core services to help meet the current crisis, Europe has put forward the COVID-19 situation as a rapid application of the core infrastructure of the European Open Science Cloud (EOSC). The Data Together group and the VODAN partners fully endorse that, but also realise that the pandemic requires a globally coordinated response. The communities that have self-organised in the VODAN IN have assigned their coordinated activities to clusters. This was also needed in practice as the interest in VODAN in the first 2 weeks after its inception and approval was quite overwhelming (Fig. 1). The clustering activity was started to ensure optimal synergy and convergence, as well as to enable easier coordination and support. We have asked early subscribers to take responsibility and coordination of subgroups addressing particular topics. The VODAN-IN will hopefully be an effective mechanism to coordinate globally dispersed and funded efforts, with co-leading participation of all continents, including Africa, to jointly rise to the challenge and ensure that what is implemented for COVID-19 is sustainable and scalable. On the other hand, it is important to manage expectations clearly and to spell out the limitations of what can be achieved. Many other COVID-19 projects will mushroom and may or may not join or benefit from VODAN-coordinated data and analytics activities, and data accessibility and analytics, even if distributed and massive, do not guarantee that we quickly find new approaches to combat the virus. However, approaching this challenge in genuine partnership around the globe is definitely needed (Fig. 2).

Such a partnership can rapidly show the enhanced feasibility, scalability and added value of a distributed learning system, with genuine and equitable data visiting around the globe, with full respect of national and institution sovereignty when dealing with sensitive citizen data. In future epidemics, we might be able to much more rapidly discern meaningful patterns in data that lead to optimal responses and policies. The VODAN community is thus responding also to the need to work closely with the EOSC to respond with FAIR data to the COVID crisis (see First ERAvsCorona Action Plan: https://ec.europa.eu/info/sites/info/files/research_and_innovation/research_by_area/documents/ec_rtd_era-vs-corona.pdf).

## Footnotes

1 VirusOutbreak Data Network (VODAN) Implementation Network: https://www.go-fair.org/implementation-networks/overview/vodan/

2 CODATA: https://codata.org

3 International Science Council: https://council.science

4 Research Data Alliance: https://www.rd-alliance.org

5 World Data system: https://www.icsu-wds.org

6 Data Together’ statement: https://www.go-fair.org/wp-content/uploads/2020/03/Data-Together_March-2020.pdf

7 Data Together COVID-19 appeal and actions: https://www.rd-alliance.org/data-together-covid-19-appeal-and-actions

8 RDA-COVID-19 Working Group: https://www.rd-alliance.org/groups/rda-covid19

9 Personal Health Train IN: https://www.go-fair.org/implementation-networks/overview/personal-health-train/

10 IN-Africa: https://www.go-fair.org/implementation-networks/overview/

11 Data Stewardship Competence Centers IN: https://www.go-fair.org/implementation-networks/overview/dscc/

12 C2CAMP IN: https://www.go-fair.org/implementation-networks/overview/c2camp/

13 Chemistry IN: https://www.go-fair.org/implementation-networks/overview/chemistryin/

14 CO-OPERAS IN: https://www.go-fair.org/implementation-networks/overview/co-operas/

15 Discovery IN: https://www.go-fair.org/implementation-networks/overview/discovery/

16 FAIR StRePo IN: https://www.go-fair.org/implementation-networks/overview/fair-strepo/
